# Genetic diversity among major endemic strains of *Leptospira interrogans *in China

**DOI:** 10.1186/1471-2164-8-204

**Published:** 2007-07-01

**Authors:** Ping He, Yue-Ying Sheng, Yao-Zhou Shi, Xiu-Gao Jiang, Jin-Hong Qin, Zhi-Ming Zhang, Guo-Ping Zhao, Xiao-Kui Guo

**Affiliations:** 1 Department of Microbiology and Parasitology, Shanghai Jiao Tong University School of Medicine, Shanghai 200025, China; 2 National Engineering Center for Biochip at Shanghai, Zhangjiang High Tech Park, Shanghai 201203, China; 3 National Institute for Communicable Disease Control and Prevention, Chinese Center for Disease Control and Prevention (ICDC, CCDC), Beijing 102206, China; 4 State Key Laboratory for Disease and Health Genomics, Chinese National Human Genome Center at Shanghai, Zhangjiang High Tech Park, Shanghai 201203, China

## Abstract

**Background:**

Leptospirosis is a world-widely distributed zoonosis. Humans become infected via exposure to pathogenic *Leptospira *spp. from contaminated water or soil. The availability of genomic sequences of *Leptospira interrogans *serovar Lai and serovar Copenhageni opened up opportunities to identify genetic diversity among different pathogenic strains of *L. interrogans *representing various kinds of serotypes (serogroups and serovars).

**Results:**

Comparative genomic hybridization (CGH) analysis was used to compare the gene content of *L. interrogans *serovar Lai strain Lai with that of other 10 *L. interrogans *strains prevailed in China and one identified from Brazil using a microarray spotted with 3,528 protein coding sequences (CDSs) of strain Lai. The cutoff ratio of sample/reference (S/R) hybridization for detecting the absence of genes from one tested strain was set by comparing the ratio of S/R hybridization and the in silico sequence similarities of strain Lai and serovar Copenhageni strain Fiocruz L1-130. Among the 11 strains tested, 275 CDSs were found absent from at least one strain. The common backbone of the *L. interrogans *genome was estimated to contain about 2,917 CDSs. The genes encoding fundamental cellular functions such as translation, energy production and conversion were conserved. While strain-specific genes include those that encode proteins related to either cell surface structures or carbohydrate transport and metabolism. We also found two genomic islands (GIs) in strain Lai containing genes divergently absent in other strains. Because genes encoding proteins with potential pathogenic functions are located within GIs, these elements might contribute to the variations in disease manifestation. Differences in genes involved in O-antigen biosynthesis were also identified for strains belonging to different serogroups, which offers an opportunity for future development of genomic typing tools for serological classification.

**Conclusion:**

CGH analyses for pathogenic leptospiral strains prevailed in China against the *L. interrogans *serovar Lai strain Lai CDS-spotted microarrays revealed 2,917 common backbone CDSs and strain specific genes encoding proteins mainly related to cell surface structures and carbohydrated transport/metabolism. Of the 275 CDSs considered absent from at least one of the *L. interrogans *strains tested, most of them were clustered in the *rfb *gene cluster and two putative genomic islands (GI A and B) in strain Lai. The strain-specific genes detected via this work will provide a knowledge base for further investigating the pathogenesis of *L interrogans *and/or for the development of effective vaccines and/or diagnostic tools.

## Background

The genus *Leptospira *comprises a heterogeneous group of saprophytic and pathogenic species belonging to the order *Spirochaetales *[1]. Pathogenic *Leptospira *spp., including *L. interrogans*, *L. kirschneri*, *L. noguchii*, *L. borgpetersenii*, *L. santarosai*, *L. weilii*, and *etc*. [2], are etiological agents of leptospirosis. They are excreted in urine of the infected animals and may penetrate the human body through skin or mucous membranes when the host contacts with contaminated water or soil [3]. Because of the wide spectrum of animal species that serve as reservoirs, leptospirosis is considered the most widely spread zoonotic disease [1].

The genus *Leptospira*, including pathogenic and saprophytic species, can be further classified into serological types, *i.e*., serogroups and serovars, defined by a cross-agglutination absorption test. The alternative genotypic classification is based on DNA hybridization and thus, the leptospires can be assigned to the species level [4-6]. However, these two classification systems are not always consistent. Strains belonging to the same serovar may belong to different *Leptospira *species and *vice versa *[2,6].

*L. interrogans *serovar Lai is a virulent serovar of serogroup Icterohaemorrhagiae, which is more likely to cause severe leptospirosis than the other serovars prevailing in China [7]. Following the determination of the complete genomic sequence of the *L. interrogans *serovar Lai strain Lai (#56601) in 2003 [8], the genome of another *L. interrogans *serovar Copenhageni strain Fiocruz L1-130 of the same serogroup Icterohaemorrhagiae was sequenced and released [9,10]. Genomic comparison of strain Lai with strain Fiocruz L1-130 revealed extensive variation in the number and distribution of insertion sequences and other genomic contents [10], which should eventually determine the unique phenotypes of each strain.

Although whole-genome sequencing is a powerful method of genetics and genomics, it is still laborious and expensive. Recently, comparative genomic hybridization (CGH) has been used to facilitate the comparison of unsequenced bacterial genomes in order to monitor the gene contents of closely related bacterial species [11-15]. Based on the genomic sequence of *L. interrogans *serovar Lai strain Lai, we constructed a microarray to compare the genomes of a number of *L. interrogans *serovars in order to clarify their genetic relationship and identify features that may serve as molecular markers to profile the serovars or genospeices, which may correspond to different levels of disease manifestation. Eleven *L. interrogans strains *that are endemic in China were analyzed. Sequences absent in *L. interrogans *were mostly confined to regions in the *rfb *gene cluster and two genomic islands (GIs) of strain Lai. The results are discussed in the context of the possible role of these regions in *L. interrogans *with respect to serovar determination and virulence.

## Results and discussion

### CGH microarray analysis and *in silico *genomic comparison of *L. interrogans *serovar Lai vs *L. interrogans *serovar Copenhageni

The genomes of two *L. interrogans *strains, Lai and Fiocruz L1-130 belonging to the same serogroup Icterohaemorrhagiae, were completely sequenced in China and Brazil respectively [8,9]. The genomic contents of these two strains were compared by CGH employing a strain Lai sequence based whole genome CDS microarray (Methods). The genomic sequences of the two strains were also compared *in silico*. CDSs of strain Lai absent in Fiocruz L1-130 were predicted by a BLASTN search, and the degree of similarity between the matching tested genomic sequence and the probe itself in terms of the length of match and the percentage sequence identity at the DNA level was expressed as H values (see Methods).

**Figure 1:**
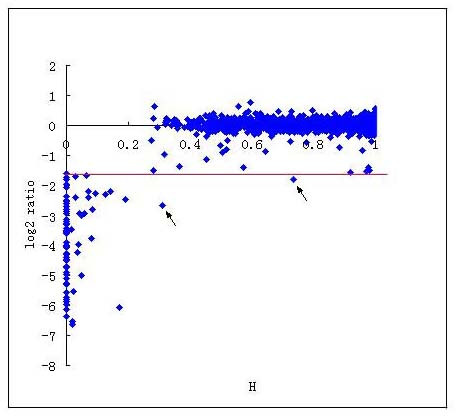
**The correlation of the level of sequence similarity determined by BLASTN (H values) and the degree of chromosomal DNA hybridization determined by microarray CGH analysis (log_2 _S/R ratio) between *L. interrogans *strain Lai and Fiocruz L1-130**. Blue dot indicates the each CDS's H value and its corresponding log_2 _ratio (S/R); red line indicates the cutoff ratio (log_2 _S/R ratio), which is -1.585.

For CGH analysis with DNA microarray slides, it is important to set an appropriate threshold to detect CDSs missing from the sample strains. In this study, we compared the H values to the results of CGH between strain Lai and Fiocruz L1-130 expressed by the normalized signal ratio of sample strain/reference strain hybridization (S/R ratio or ratio, hereafter, Fig. 1). It is clear that the plot of H values of each CDS versus its corresponding log_2 _S/R ratio values was divided into two groups defined by the apparent cutoff values of H and the log_2 _ratio. We may define the cutoff H value as 0.2 because any CDS is either absent or significantly divergent in strain Fiocruz L1-130 from that of strain Lai, when H ≤ 0.2 (total 59 CDSs, among them, 37 with H = 0, i.e., total deletion). For the same token, if the H value of a CDS is larger than 0.2, it can be considered a conserved gene. We may also draw the cutoff S/R ratio for hybridization as 0.33 (log2 ratio = -1.585) because this value divides the CDSs into two categories almost identical to that of H = 0.2, except two data points (two dot arrowed in Fig. 1), which is the minimum among all the other possible cutoff values  (Table 2). In other words, although, for unknown reasons, there were genes with unexpected signal ratios in groups IV and VI, the numbers of such genes were relatively low (1 of 89 genes and 1 of 313 genes) and with this cutoff, we may have the minimal 3.3% false positives (2/61) and zero false negatives (0/3131) among all the other choices (refer to Table 2).

**Table 2:** Distribution of the sample/reference (S/R) signal ratios of strain Lai CDSs hybridized to the chromosomal DNA of Fiocruz L1-130 by H values derived from *in silico *DNA sequence similarity comparison of the two strains*

Group	H value	No. of CDSs in DNA array	No. of CDSs at a S/R ratio of:	
			
			≤ 0.33	> 0.33
I	≥ 0 and < 0.1	55	55	0
II	≥ 0.1 and < 0.2	4	4	0
III	≥ 0.2 and < 0.3	4	0	4
IV	≥ 0.3 and < 0.5	89	1	88
V	≥ 0.5 and < 0.7	246	0	246
VI	≥ 0.7 and < 0.9	313	1	312
VII	≥ 0.9 and ≤ 1	2481	0	2481

Total	≥ 0 and ≤ 1	3192	61	3131

* Among the total of 3528 CDSs array, there were 336 invalid data for low quality signal of hybridyzation derived from all of the CGH experiments. That makes the total 3192 CDSs listed in the table.

An S/R ratio of -1 on the log_2 _scale was frequently used in previous studies [11,15]. However, for this study, with the threshold value of -1, four conserved genes with H value more than 0.9 would be designated as absent in strain Fiocruz L1-130. Therefore, a threshold log_2 _ratio value of -1.585 (S/R ratio = 0.33) determined by the statistical correlation between the S/R ratio and the genomic sequence similarity (H value) of *L. interrogans *strain Lai and strain Fiocruz L1-130 is more appropriate than the artificial value of -1 for detecting the absent/divergent CDSs for the *L. interrogans strain Lai whole genome CDS microarray-based CGH *studies.

### Overview of the microarray analysis

**Figure 2:**
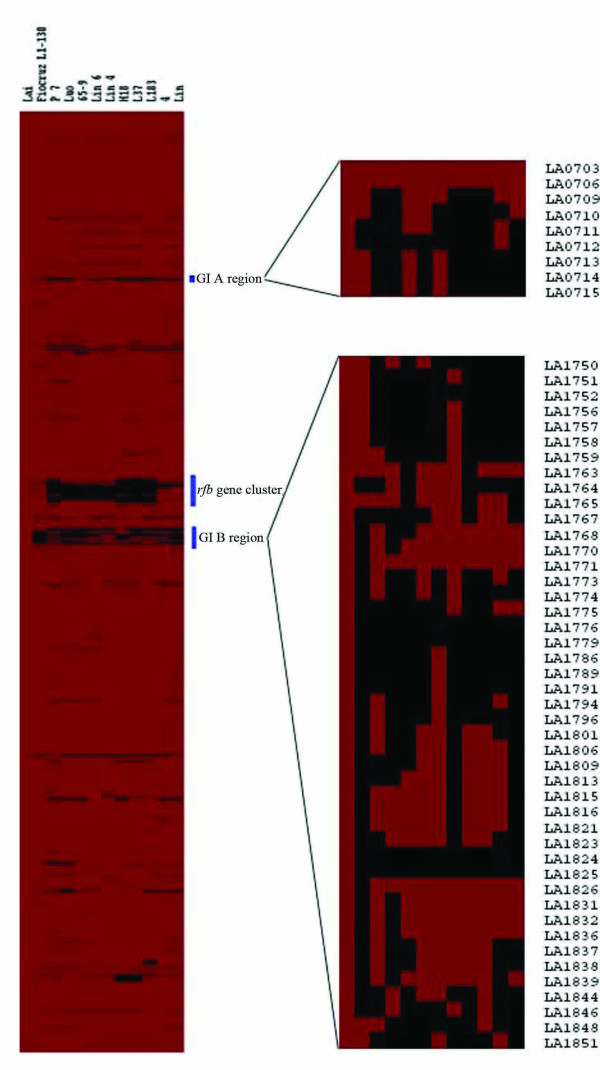
**Genetic diversity of *L. interrogans *strains detected by microarray CGH analysis**. Red and black areas denote the genes that are present/conserved and absent/divergent respectively, and the genes are arranged in order of LA and LB numbers from LA0001 at the top to LB367 at the bottom. The columns represent the strains analyzed and are labeled on top of the left panel. Blue bars indicate the *rfb *locus and two GI regions. Zoomed images of the right panel represent the two genomic island-like regions: GI A and GI B region. The genes in these regions are defined by their LA numbers.

The genomic contents of the 11 *L. interrogans *strains were analyzed by CGH using the CDSs encoded by the genome of strain Lai as reference. The results are shown in Fig. 2 and Additional file 1. Of the 3,528 CDSs spotted on the microarray slides, 275 were considered absent from at least one of the *L. interrogans *strains tested. These CDSs accounted for 5.8% of all the CDSs of strain Lai annotated [8] or 7.9% of the CDSs spotted on the slides. With invalid data excluded (Methods), the remaining 2,917 CDSs were likely conserved in all the strains used in this study. There were differences in the numbers of absent CDSs for different strains, ranging from 61 in strain Fiocruz L1-130 to 161 in strain P7 (Table 1).

**Table 1:** Bacterial strains used in this study and number of absent CDSs in each strain detected by CGH

Serogroup	Serovar	Strain	Source	Source-Animal	No. of absent CDSs
Icterohaemorrhagiae	Lai	Lai	Sichuan Province, China	Human	0
Canicola	Canicola	Lin	Guangdong Province, China	Human	110
Pyrogenes	Pyrogenes	4	Guangdong Province, China	Human	90
Autumnalis	Autumnalis	Lin 4	Zhejiang Province, China	Human	98
Australis	Australis	65-9	Fujian Province, China	Horse	122
Pomona	Pomona	Luo	Fujian Province, China	Human	151
Grippotyphosa	Linhai	Lin 6	Zhejiang Province, China	Human	98
Hebdomadis	Hebdomadis	P 7	Sichuan Province, China	Human	161
Bataviae	Paidjan	L 37	Guangxi Province, China	Human	159
Sejroe	Wolffi	L 183	Yunnan Province, China	Human	137
Sejroe	Haemolytica	H 18	Yunnan Province, China	Human	144
Icterohaemorrhagiae	Copenhageni	Fiocruz L1-130	Salvador, Brazil	Human	61

Sixteen of the 275 absent CDSs were chosen for confirmation tests with PCR amplification in 12 *L. interrogans *strains. Only 4 reaction results did not match the CGH results among the 192 PCR reactions. Moreover, we also validated the CGH results by comparing them to the publicly available sequence variation data of the *rfb *loci from five serovars of *L. interrogans *(Canicola, Pyrogenes, Autumnalis, Australis and Pomona) [16] employing the BLASTN *in silico *hybridization method. The two results were well matched and in conclusion, our CGH results are reliable and reasonable.

The distribution of the absent genes indicated that majority of the absent/divergent genes were clustered in three regions (Fig. 2). The *rfb *gene cluster (from LA1576 to LA1672) was previously described [8]. The other two regions were referred as GI A (from LA0702 to LA0717) and GI B (from LA1747 to LA1851) respectively. The rest absent genes were scattered over the genome.

The proportions of the absent CDSs with respect to their functional category are shown in Table 3. Genes encoding fundamental cellular functions were relatively conserved (absent genes only counted less than 5% of the corresponding categories), such as translation (2.82%), energy production and conversion (1.31%, the lowest rate of gene absent for all categories), as well as transport and metabolism for lipid and inorganic ions (both less than 2%). In contrast, genes in 5 functional categories including cell cycle control and defense mechanisms have over 10% of the CDSs absent. Approximately 16.53% of the genes assigned to cell wall biogenesis including sugar biosynthetic enzymes and outer membrane efflux proteins were absent from most of the *L. interrogans *strains analyzed. Approximately 13.19% of the genes assigned to carbohydrate transport and metabolism and 14.52% of those assigned to secondary metabolites biosynthesis, transport and catabolism were also missing. One must clarify that genes of the last category mainly encode methyltransferases, which are likely involved in sugar modification. Thus, it is not surprising that most of the absent genes in these three categories (82% absent genes of cell wall biogenesis, 95% absent genes of carbohydrate transport and metabolism and 78% absent genes of secondary metabolite biosynthesis, transport and catabolism) were located in the O-antigen (*rfb*) locus.

**Table 3:** Distribution of strain Lai CDSs and CGH analysis identified absent CDSs in tested strains by functional classes

Category*	No. of CDSs in strain Lai	No. of CDSs absence^#^	% of absent CDSs
Translation	177	5	2.82
Transcription	154	10	6.49
Replication, recombination and repair	250	15	6.00
Cell cycle control, mitosis and meiosis	63	8	12.70
Defense mechanisms	51	6	11.76
Signal transduction mechanisms	254	8	3.15
Cell wall/membrane biogenesis	236	39	16.53
Cell motility	120	6	5.00
Intracellular trafficking and secretion	73	5	6.85
Posttranslational modification, protein turnover, chaperones	121	4	3.31
Energy production and conversion	153	2	1.31
Carbohydrate transport and metabolism	144	19	13.19
Amino acid transport and metabolism	218	7	3.21
Nucleotide transport and metabolism	62	2	3.23
Coenzyme transport and metabolism	116	3	2.59
Lipid transport and metabolism	114	2	1.75
Inorganic ion transport and metabolism	145	2	1.38
Secondary metabolites biosynthesis, transport and catabolism	62	9	14.52
General function prediction only	504	49	9.72
Function unknown	228	15	6.58

* Functional categories of strain Lai CDSs were based on information from the GenBank database [67].

^# ^Some of the CDSs were not include in any of the categories, while some other CDSs may belong to more than one category. Therefore, the sum of the absent genes for individual category does not equal to the total number of the absent CDSs

### Structure and function of the genomic islands

Three bacteriophages of the saprophytic *L. biflexa *were first isolated by Saint Girons et al. [17]. These bacteriophages do not infect representative species of pathogenic leptospires. However, evidence for horizontal transfer of DNA among *L. interrogans *(*sensu lato*) came from studies of the intervening sequences found within the 23S rRNA gene [18] and from the finding that the leptospiral lipopolysaccharide biosynthetic locus (*rfb*) is located in a genomic island that was probably acquired through horizontal transfer from gram-negative source(s) [19,20].

**Figure 3:**
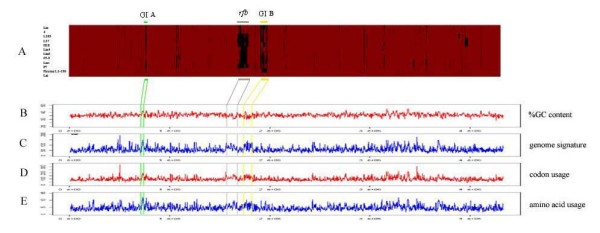
**Comparative analysis of the diverged *L. interrogans *genomes observed by CGH with respect to their signatures defining genomic islands**. (**A**) The presence/absence CDSs of all tested strains comparing to that of strain Lai ordered from LA0001 to LB367. Red and black areas denote the presence and absence of genes respectively. The blue bar indicates the GI A region, the grey bar indicates the *rfb *locus, and the yellow bar indicates the GI B region. These 3 regions are specifically drawn to the following sketches indicating the variations in strain Lai for properties of possible genomic islands: CG content (**B**), genome signature (**C**), codon usage (**D**), and amino acid usage (**E**).

Three regions with large sets of missing CDSs detected by CGH in the 11 *L. interrogans *strains presented some characteristics of laterally transferred genomic elements. Besides the *rfb *gene cluster, the GC content, genome signature, as well as the codon and amino acid usage bias of the other two missing regions were analyzed along with the chromosomal DNA sequence of strain Lai (Fig. 3) [21] to verify their GI characteristics.

The first putative genomic island (GI A) is a ~28-kb-long segment of DNA encompassing CDSs from LA0702 to LA0717. It begins with an insertion sequence IS*lin*1 and a transposase (LA0702) [9], and ends with an IS3 and a transposase gene (LA0717) [9]. This region presents many characteristics expected for a typical GI [21-23]: (1) higher GC content, (2) altered codon preference, (3) different amino acid usage pattern, and (4) genes encoding transposases at the ends (CDSs LA0702 and LA0717).

Among the 16 CDSs included in this region, there are 8 CDSs without any significant homology to genes in the GenBank database. Besides the 2 proximal genes both encoding transposases, there are 4 other genes encoding a transposase, an integrase, a molybdate metabolism regulator and a lipoprotein, respectively.

There are two genes likely related to pathogenesis. LA0705 was found to contain an LRR (Leucine-rich repeats), which is characteristic for a diverse array of proteins providing a versatile framework for protein-protein interaction [24]. It is particularly interesting to notice that, all of the bacterial LRR proteins that have been well characterized so far, including those from *Listeria monocytogenes *[25], *Streptococcus *[26], *Yersinia pestis *[27], *Salmonella typhimurium *[28] and *Shigella flexneri *[29], are implicated in virulence. It was shown to be a major virulence factor in *L. monocytogenes *presumably functioning as triggering engulfment of the bacterium after specifically interacting with cell-surface receptors [25]. On the basis of their sequence similarity, the probable pathogenic function of this gene in *L. interrogans *serovar Lai is worth to be further explored.

Another CDS, LA0706, shares amino acid homology with hemin receptor of gram-negative bacteria involved in the acquisition of iron from hemin and hemoglobin, such as the ChuA of *Escherichia coli* O157:H7 [30], HemR of *Yersinia enterocolitica *[31], ShuA of *Shigella dysenteriae *[32], and HgpB of *H. influenzae *[33]. Its contribution to bacterial virulence was proven in uropathogenic *E. coli*[34] and the heme scavenging function of the hemin receptor ChuA is speculated to depend on the activity of α-hemolysin, which gains access to the intracellular heme reservoir [35]. Since 9 hemolysins were confirmed in strain Lai [36,37], one may speculate that the coupling of hemolysis with heme utilization could serve as an effective iron acquisition strategy during the progression of strain Lai infection.

The second large GI (GI B) spans almost 83 kb, from LA1747 to LA1851. It is not inserted at the 3' end of a tRNA gene and it bears no significant variations in its GC content. However, it does have all the expected properties of a typical GI in other aspects. It begins with an insertion element IS*1501 *and a transposase [9], and ends with a transposase. This island also has large numbers of CDSs encoding proteins with unknown functions, but several of others are homologous to the bacteriophage-encoded proteins (LA1833, LA1835 and LA1836) and integrases (LA1768 and LA1811). This indicates that phage-mediated integration events may be involved in the acquisition of this island.

Of the 45 CDSs in the GI B region spotted on the microarray, majority of them were missing from the strains tested (Fig. 2). The pattern of the absent genes seemed highly mosaic. Further concerning the very low level of variation in its GC content and the presence of multiple transposases, it may suggest that the GI B region is likely a site experienced extensive insertion, excision and recombination and it could be acquired from species with G+C content similar to that of *L. interrogans *or that the base composition of the acquired DNA have gradually adapted to the host genome.

It is particularly interesting that Fiocruz L1-130 lacks the whole GI B segment except 11 genes located at the two ends of this region. This missing region covers a 54-kb DNA segment specific to strain Lai (from LA1768 to LA1847) [10]. Recently, Bourhy and his colleagues named this 54-kb DNA region LaiGI I and demonstrated it can be excised from the chromosome to form a replicative plasmid [38]. They also observed imprecise excision of LaiGI I in *L. interrogans *serovar Lai. This finding may further support the mosaic character of the GI B region detected in different strains of *L. interrogans*, which is larger than and covers the whole segment of LaiGI I.

The GI B also contains genes encoding putative regulators. For example, the AraC family transcriptional regulator gene (LA1770) has been shown to regulate diverse bacterial functions including sugar catabolism, response to stress and virulence [39-43].

Horizontal gene transfer plays an important role in the evolution of different bacterial pathotypes [22]. The two putative GIs found in strain Lai contained many divergent genes with several features of pathogenicity and metabolic islands. Because these GIs are largely missing in other pathogenic *L. interrogans *spp., they may not encode genes essential for pathogenesis but might contribute, to certain extent, the severe pathogenic properties of serovar Lai infection [7].

### Structure and function of the *rfb *gene cluster

Leptospiral LPS plays critical roles in both pathology and immunity during the course of leptospirosis and forms the basis for serological classification of *Leptospira *spp. [1,44-46]. The O-antigens are synthesized by a set of enzymes encoded by the *rfb *gene cluster in addition to a few genes scattered over the whole chromosome [8]. The nucleotide sequence of the strain Lai *rfb *locus spanning LA1576- LA1672 comprises 103 kb [8]. CGH analysis revealed that although the *rfb *gene cluster is frequently absent from all strains tested except Fiocruz L1-130, its 3'-proximal end is conserved, which spans from LA1658 through to LA1672. In contrast, the genetic layout at the 5'-proximal end is more variable. Because the genes located in this segment of strain Lai (and Fiocruz L1-130) were predicted to encode glycosyltransferases and enzymes catalyzing sugar activation, the genetic variations of this segment is likely to cause the variations in LPS composition/structure of the tested strains. These results confirmed previous reports that the genetic basis for serological differences among leptospiral SVs were related to the presence of specific sugar-biosynthetic or -modifying genes in their respective *rfb *loci [16,46].

**Figure 4:**
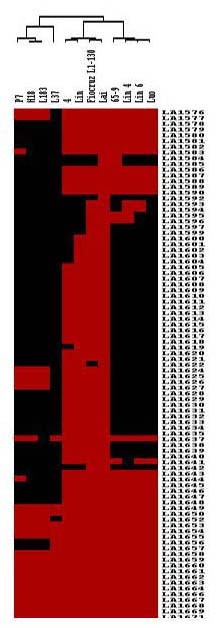
**Cluster analysis of *L. interrogans *serovar Lai *rfb *genes**. The subset of 89 *rfb *genes in microarray was used to generate a dendrogram of strains based on the presence or absence of the genes. The strains were grouped by average hierarchical clustering using the CLUSTER program and the output displayed using the TREEVIEV program. Shown at the top is the unrooted tree for the relationship of the serovars. Red and black areas denote the presence and absence of genes respectively.

In addition, comparison of the *rfb *loci of strains Lai and Fiocruz L1-130, both belong to the same serogroup, Icterohaemorrhagiae, revealed only minor gene diversity. Hierarchical clustering of the CGH data based on the 89 *rfb *genes further revealed the phylogenetic relationship among different strains (Fig. 4). The unrooted (uneducated) tree revealed that strains Lai and Fiocruz L1-130 were clustered together but showed relatively low correlation to other strains in this study. Interestingly, strains L183 and H18 (both belong to the same serogroup, Sejroe) were also clustered together. This result implies that the compositions of the *rfb *locus genes from strains of the same serogroup are likely more similar to each other than those of different serogroups. Although strains belonging to different serogroups were also found to fall under the same node, such as strains 4 and Lin, and strains Lin4, Lin6 and Luo; no strains belonging to the same serogroup were separated into two or more different nodes. Due to the lack of comprehensive sequence information for the *rfb *loci of all the strains tested, this result may lie in incapable of identifying *rfb *genes present in the tested strain but absent from the strain Lai-based microarray.

Because of the key role of leptospiral LPS in pathology, immunity and taxonomy, the continued investigation of LPS biosynthetic genes in other serovars, particularly the strain-specific and/or missing regionsof the *rfb *loci is important. Conserved sequences flanking missing CDSs identified by the CGH analysis for different serovar strains might serve as appropriate primer candidates for amplifying strain-specific regions, which could eventually be useful for rapid identification and isolation of characteristic genomic segments (genes or gene clusters) corresponding to leptospiral serogroups and serovars.

### Genes associated with immunity

Human vaccines composed of inactivated whole bacterial cell or outer membrane envelope are available in some countries to prevent leptospirosis [47,48]. However various kinds of serovar specificity limited the efficacy of protection against different pathogenic leptospires [49,50]. A major focus of research for the prevention of leptospirosis is to identify proteins conserved among pathogenic leptospires, which may generate cross-protection against strains of various serovars [51-55]. In addition to the complete genomic sequence information for pathogenic bacteria, CGH analysis was useful as one of the approaches based on reverse vaccinology [56] for screening vaccine candidates against leptospirosis.

Genes that are highly conserved over a broad range of strains could be useful for the development of a protein-based vaccine capable of protecting hosts against most of the pathogenic serogroups of *L. interrogans *in China. Our results showed that 24 putative lipoprotein or outer membrane proteins were not conserved among the strains tested [see Additional file 2]. However, previous report indicated that OmpL1 and LipL32 were highly conserved among the main endemic strains of *L. interrogans *in China [57]. This CGH analysis not only confirmed these results but further identified additional conserved leptospiral protein antigen candidates, such as LipL41 and immunoglobulin-like proteins, and they have been shown to elicit protective immunity in animal models [58-60].

### Virulence factors

The primary lesion caused by leptospiral infection is damaging to the endothelium of small blood vessels, leading to hemorrhage and localized ischemia in multiple organs [6]. Potential virulence factors such as hemolysin, protease and ankyrin-like proteins are suggested for pathogenesis of leptospirosis [10]. The CGH results showed that most of the potential virulence factors were conserved among all strains. The *L. interrogans *strain Lai microarray included the 7 genes coding for hemolysins [36,37], of which, 5 (LA0327, LA0378, LA1650, LA3050 and LA3937) were conserved among all strains tested. Two hemolysin genes (LA1027 and LA1029) were absent from strain 65-9. Ankyrin repeats were found in numerous proteins mediating specific protein-protein interactions [61]. Genes encoding ankyrin-like proteins were found in bacterial genomes located in close proximity to genes encoding proteins involved in either nutrient acquisition and uptake or tolerance/resistance to antibiotics, starvation or oxidative stress [62,63]. The microarray used for this CGH analysis included the 11 ankyrin-like protein encoding genes. These genes were conserved in all strains except LA2263, which was absent from strain 65-9. The gene encoding collagenase was also conserved in all strains tested.

## Conclusion

*L. interrogans *serovar Lai strain Lai whole genome CDSs microarray based CGH analysis revealed extensive similarities in gene content among *L interrogans *strains of different serovars endemic in China. We discovered that 2,917 of the 3,528 CDSs represented on the microarray were present/conserved in any of the 11 given *L interrogans *strains. Only 275 CDSs were absent when compared to *L interrogans *strain Lai. Most of these strain specific genes are focused on 3 genomic island-like loci. Of which, two GIs (GI A and GI B) had several features as pathogenicity and metabolic islands. Both of them contain many divergent genes, which may contribute to differences in disease manifestation. Differences in the genes involved in O-antigen synthesis largely focused on the third genomic island-like *rfb *locus were also identified in strains belonging to different serogroups, which will open new avenues for the development of rapid typing tools by analyzing serovar-specific genes. Although strain-specific genes may result from genetic drift, some of them are likely to encode proteins adapted to genetically diverged hosts or factors contributed to different disease outcomes. Given the small sample size and lack of clinical information for many of the strains, we cannot correlate these specific genes with particular disease outcomes. However, The strain-specific genes presented by this work will form the basis for further investigation of the pathogenesis of *L interrogans *and will be useful for data-mining aiming at future development of effective vaccines or diagnostic means.

## Methods

### Bacterial strains and culture conditions

The *L. interrogans *strains used in this study are listed in Table 1. Strain Fiocruz L1-130 belongs to serovar Copenhageni, one of the prevalent serovars in Brazil. *L. interrogans *serovar Lai strain Lai was encoded #56601 by National Institute for the Control of Pharmaceutical and Biological Products (NICPBP) of China and was maintained by the Chinese Center for Disease Control and Prevention (CCDC) with other prevalent pathogenic leptospiral strains in China, isolated from human or horse. The genomic DNA of strain Fiocruz L1-130 was kindly provided by the Centro de Pesquisas Goncalo Moniz. Strains were grown in liquid Ellinghausen-McCullough-Johnson-Harris (EMJH) medium [64] at 28°C under aerobic conditions and collected at a density of about 10^8 ^bacteria per ml. Bacterial genomic DNA was purified using a Bacteria Genomic DNA kit (Huashun Co.) according to the manufacturer's instructions.

### Construction of *L. interrogans *CDS microarray

Annotation of the *L. interrogans *serovar Lai strain Lai genome identified 4727 CDSs (accession number GB: AE010300 for CI and GB: AE010301 for CII). Among them, very short CDSs (less than 250 bp) and CDSs highly homologous to each other at the nucleotide levels were all excluded and a total of 3,528 annotated CDSs were selected for microarray fabrication. PCR primers were designed using Primer3. All primers were synthesized by Dgbio Co. *L. interrogans *serovar Lai strain Lai genomic DNA was used as the template for PCR amplification. The thermal cycle parameters were 30 sec denaturation at 94°C, 45 sec annealing at 55°C and 1.5 min elongation at 72°C for 35 cycles. Amplified products were checked on agarose gels to verify their size and quantity, and were scored as successful if a single product of the expected mobility was detected. Amplified products were ranged from 250 to 1200 bp. The final array consisted of 3528 CDSs (74.6% of annotated CDSs). The PCR products were then purified using 96-well Multiscreen PCR plates (Millipore) following the user manual instructions. The purified DNAs were air-dried at 65°C, and were re-suspended in 30 μl of 50% DMSO. Final concentration of the spotting sample is 250 ng/μl.

### Microarray printing and processing

The PCR products (250 ng/μl) were spotted in triplicate on to glass slides (FullMoon Biosystem) coated with polylysine, following the standard protocol developed by P. Brown, Stanford, CA [65]. The DNA of the human β-actin gene and 50% DMSO were included in the microarray design as internal control elements. The spotter and software used were from GeneMachines (Omnigrid and Gridder 2.0).

### Microarray, labeling and hybridization

The genomic DNA of *L. interrogans *serovar Lai strain Lai was used as reference DNA in a double-fluorescence hybridization. Genomic DNAs from the reference strain Lai and other test strains were sonicated to fragments, ranging from 250 bp to 2000 bp in lengths. These DNA fragments were used as templates for the direct incorporation of fluorescent nucleotide analogs (Cy3- and Cy5- dCTP respectively) (Amersham Biosciences Co.) by a randomly-primed polymerization reaction. In brief, 3 μg of genomic DNA was labeled with 6 μg of random nonamers (Takara), 25 U of the Klenow fragment (New BioLab) and 1 nmol of Cy3- or Cy5-dCTP at 37°C for 3 h. Probes were purified by a QIAquick Nucleotide Removal Kit according to the manufacturer's instructions (Qiagen). Purified DNA probes were dried and finally resuspended in 8 μl of sterilized distilled water. The labeled DNA sample was combined with 20 μl formamide, 7.5 μl 20 × SSC, 0.3 μl 10% SDS, 1 μl 10 mg/ml salmon sperm DNA (Life Technologies), denatured for 3 min at 99°C, and applied to the microarray slide, which was then covered with a 24 × 50 mm glass coverslip. The labeled DNA was hybridized to the DNA microarray in a hybridization chamber at 42°C for 16 h. When the hybridization was complete, the slides were washed at 55°C with 1 × SSC containing 0.2% SDS for 10 min and then at 55°C with 0.1 × SSC containing 0.2% SDS for 20 min, and finally at room temperature with 0.1 × SSC for 3 min. The last step was conducted twice. The slides were immediately dried and scanned for fluorescence intensity using a GenePix 4000B microarray scanner (Axon Instruments), and the results were recorded in 16-bit multi-image TIFF files. Competitive hybridization was conducted twice for each strain. In the first experiment, the strain Lai reference DNA and the sample DNA were labeled with Cy3 and Cy5, respectively. In the second hybridization, the dyes for labeling were interchanged.

### Data analysis

The signal intensity of each spot in the microarray was quantified using GenePix Pro 4.0 (Axon Instruments) software. Additional data analyses were conducted by the computer software programs Microsoft Excel and GeneSpring 5.0.2 (Silicon Genetics). The data were filtered so that spots with the reference (strain Lai) signal lower than background plus 2 standard deviations of background were discarded. Signal intensities were corrected by subtracting the local background. Sample/reference (S/R) ratios of signal intensity were calculated and were transformed to logarithm base 2. The ratios were normalized by taking the median log_2 _ratios of all spots as 0. To determine the final value for each CDS tested, the median value was calculated from three log_2 _ratios obtained from 1 DNA microarray slide. In addition, to ensure only high quality data were used for analysis, spots that gave invalid results in one strain tested were considered as invalid results in other tests and thus, discarded. This allowed for the retrieval of 3,192 spots. CDSs were considered absent/divergent if the final ratios of signal intensities were both less than -1.585 on the log_2 _scale in two dye-interchange experiments.

### Genomic comparison of the *L. interrogans *serovar Lai and *L. interrogans *serovar Copenhageni strains *in silico*

Each nucleotide sequence of the CDSs assigned in the genome of strain *L. interrogans *serovar Lai (accession number GB: AE010300 for CI and GB: AE010301 for CII) was used as a query for a homology search with BLASTN against the genome sequence of the *L. interrogans *serovar Copenhageni (accession number AE016823, AE016824). The region with the highest score for each query was retrieved and classified by the H value. This homology score was proposed by Fukiya et al. [66] and reflects the degree of similarity between the matching test genome sequence and the probe itself in terms of the length of match and the percentage sequence identity at the DNA level. For each query, the H value was calculated as follows: [(length of highest-score region) × (identities of hit shown in BLASTN)]/(length of query sequence). If there was no sequence with a BLASTN E value less than 0.01, the query CDS was judged to be absent from the *L. interrogans *serovarCopenhageni genome, and its H value was 0. Queries for genes that were probably absent gave low H values. Therefore, H belonged to the set [0, 1]. The H value indicated how closely the corresponding sequence of *L. interrogans *serovar Copenhageni resembled the *L. interrogans *serovar Lai query CDS in terms of length and sequence identity.

### PCR validation

Sixteen genes were randomly chosen from strain-specific genes to verify the CGH results. Primer sequences are in Additional file 3. For each gene, PCR was performed in 12 *L. interrogans *strains. The parameters for amplification were as follows: 95°C for 3 min; 30 cycles of 94°C for 30 sec, 55°C for 30 sec, and 72°C for 1 min; and a final extension cycle of 72°C for 5 min. PCR products were run on agarose gels to confirm the presence of a band of the expected size.

## Authors' contributions

PH, YYS and XKG designed the research project. YYS and YZS constructed the microarray. PH, YYS and ZMZ completed the CGH. PH and JHQ carried out the data analysis. PH and XKG drafted the manuscript. XGJ and GPZ participated in the design of the study and helped to draft the manuscript. All authors contributed to the writing and preparation of the manuscript. All authors read and approved the final manuscript.

## Supplementary Material

Additional file 1 CGH data file of *L. interrogans*. Click here for file

Additional file 2 Distribution of divergent genes encode surface-exposed proteins among the strains tested. Click here for file

Additional file 3 Oligonucleotide primers used to confirm the CGH results. Click here for file
