# Supplementary material for: Genetic diversity among major endemic strains of Leptospira interrogans in China
Source: BMC Genomics. 2007 Jul 1;8:204. doi: 10.1186/1471-2164-8-204 (PMC1936430; doi:10.1186/1471-2164-8-204)
Supplement: Additional file 2 — Distribution of divergent genes encode surface-exposed proteins among the strains tested. [file 1471-2164-8-204-S2.doc]

Additional file 2: Distribution of divergent genes encode outer membrane surface proteins among the strains tested

| ID | annotation  annotation | Lai | Fiocruz L1-130 | Lin | 4 | Lin 4 | 65-9 | Luo | Lin 6 | P7 | L37 | L183 | H18 |
| --- | --- | --- | --- | --- | --- | --- | --- | --- | --- | --- | --- | --- | --- |
| LA0416 | Putative lipoprotein | +^a^ | + | - | - | - | - | - | - | - | - | + | - |
| LA0426 | Putative lipoprotein | + | + | + | + | + | + | + | + | - | + | + | + |
| LA0492 | LipL36 | + | + | - | - | - | - | + | - | + | + | - | - |
| LA0558 | Putative lipoprotein | + | + | - | - | - | - | + | - | + | - | + | - |
| LA0710 | Putative lipoprotein | + | + | + | - | - | + | - | + | - | - | - | - |
| LA0715 | Putative lipoprotein | + | + | - | - | + | + | - | - | - | - | - | - |
| LA0905 | Putative lipoprotein | + | + | + | - | + | + | + | + | + | + | + | + |
| LA0962 | Putative lipoprotein | + | + | + | + | + | + | - | + | - | + | + | + |
| LA1467 | Putative lipoprotein | + | + | + | + | + | + | + | + | + | - | + | + |
| LA1759 | Outer membrane protein | + | + | - | - | - | - | - | - | + | - | - | + |
| LA2022 | Putative lipoprotein | + | + | - | + | + | + | - | + | - | + | + | - |
| LA2443 | Outer membrane protein | + | + | - | - | + | - | - | + | - | + | + | + |
| LA2444 | Outer membrane protein | + | + | - | + | + | - | - | - | - | - | + | + |
| LA2972 | Putative lipoprotein | + | + | + | - | + | + | + | + | + | + | + | + |
| LA3262 | Putative lipoprotein | + | + | + | - | + | + | + | + | + | + | + | + |
| LA3339 | Putative lipoprotein | + | + | - | + | + | + | - | + | - | + | + | + |
| LA3340 | Putative lipoprotein | + | + | - | + | + | + | - | + | - | + | + | + |
| LA3724 | Putative lipoprotein | + | + | + | + | + | + | + | - | + | - | + | - |
| LA3726 | Putative lipoprotein | + | + | + | + | + | + | + | + | + | - | + | - |
| LA3730 | Putative lipoprotein | + | + | + | + | + | + | + | + | + | - | + | - |
| LA3735 | Putative lipoprotein | + | + | + | + | + | + | + | + | + | - | + | - |
| LA3881 | Outer membrane protein | + | + | - | - | - | - | - | - | - | - | + | - |
| LA4135 | Putative lipoprotein | + | + | + | + | + | + | - | + | + | + | + | + |
| LA4141 | Putative lipoprotein | + | + | - | + | - | + | - | + | - | + | + | + |

^a^+, present/conserved genes; -, absent/divergent genes.
