# Supplementary material for: Genetic diversity among major endemic strains of Leptospira interrogans in China
Source: BMC Genomics. 2007 Jul 1;8:204. doi: 10.1186/1471-2164-8-204 (PMC1936430; doi:10.1186/1471-2164-8-204)
Supplement: Additional file 3 — Oligonucleotide primers used to confirm the CGH results. [file 1471-2164-8-204-S3.doc]

Additional file 3: Oligonucleotide primers used to confirm the CGH results

| Gene ID | Sense primer (5’-3’) | Antisense primer (5’-3’) |
| --- | --- | --- |
| LA0640 | GTTTTATCATCGGTTTTCGG | ATACTTCGGTTGTTCGATGG |
| LA0662 | CGTAGCATTCCTATCCGTCT | GATCGGACTGTTCTTTGGAC |
| LA0709 | GATACAAGCAACTTGGGGAC | CCAGTTCCCTTTTGTTTCAC |
| LA0710 | GTGACAAGAAAGCGGAAGAT | ACCCGGATGACTACTCTCAA |
| LA0711 | CCGAAGGTAGTGGAAGAGAA | TATCCCCAAAGATTTGAACC |
| LA0712 | ACGGACAAGTATGCCTTACC | CCCTTTGACTCTTTCGTGTT |
| LA0713 | TGGAAATGCGAGTTTAGTCA | CGCAACTCATTCAACTTTTG |
| LA0715 | GGATGTGAAACGAAACATCA | ACTGGAATAACAGCTTTCGG |
| LA1832 | CCTACTTCTTGCCCTCCGTTAT | CCTTTTCTGTTCGATTCTTTGACT |
| LA1836 | TGACGACGACCTAAAACTGA | AGAAGCCAAGAAAACGAAGA |
| LA1837 | ATCAAAGGTGGAACTGGAAA | TGTGGAAAATGGAATAGGCT |
| LA1838 | CTTCTCGGTTCCAATGAAAT | TTTCTCGGCAAGTTCTTCTT |
| LA1839 | GTGGAGTTTTAGCGGAAAAG | CTTTGGGGATGGCTATAAAA |
| LA1848 | AACGAAGGAGTTTGTGAAGGATT | GCTTTCTTGAGCCAACGAGTAT |
| LA1851 | AAAGAACATCTACTACATTTGCCG | GCTGCTGTAATAAGAGCGAATGA |
| LA2681 | ATTCGACTCCCAATGTCAAT | TCGTCGAACCCTTAGATGAT |
